# Supplementary material for: Dynamics of extrachromosomal circular DNA in rice
Source: Nat Commun. 2024 Mar 18;15:2413. doi: 10.1038/s41467-024-46691-0 (PMC10948907; doi:10.1038/s41467-024-46691-0)
Supplement: Supplementary file 1 — Supplementary Information [file 41467_2024_46691_MOESM1_ESM.pdf]

Dynamics of extrachromosomal circular DNA in rice

Zhuang *et al.*

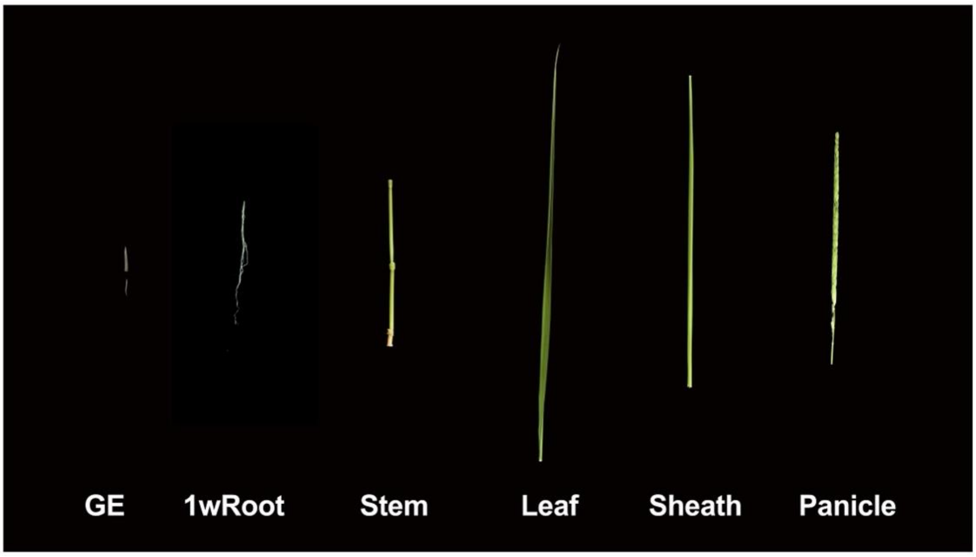

Supplementary Fig. 1. Rice tissue samples for Circle-seq sequencing. This figure depicts images of rice tissue samples utilized for Circle-seq sequencing, representing distinct tissue types: Germinated embryo (GE), Root (1-week-old, 1wRoot), Stem, Leaf, Leaf sheath, and Panicle.

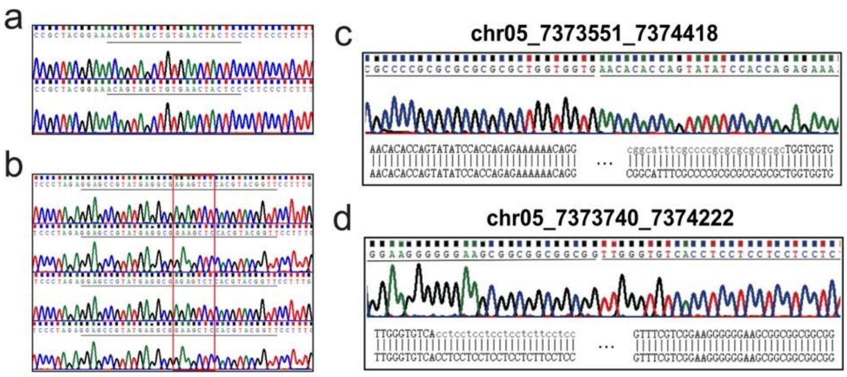

Supplementary Fig. 2. Characterization of eccDNA by Sanger sequencing. (a) Sequencing chromatograms of (chr12:20609533-20610693). (b) Sequencing chromatograms of eccDNA2 (chr12:20499833-20500504). The underlined sections denote one of two direct repeats at the genomic location's extremities. Each electrophoresis band reflects two isoforms of the eccDNA2 PCR product, with discrepancies highlighted by the red rectangle. (c) and (d) Sanger sequencing results of the PCR products corresponding to two different eccDNAs, demonstrating distinct nucleotide sequences that confirm the existence of separate eccDNA entities.

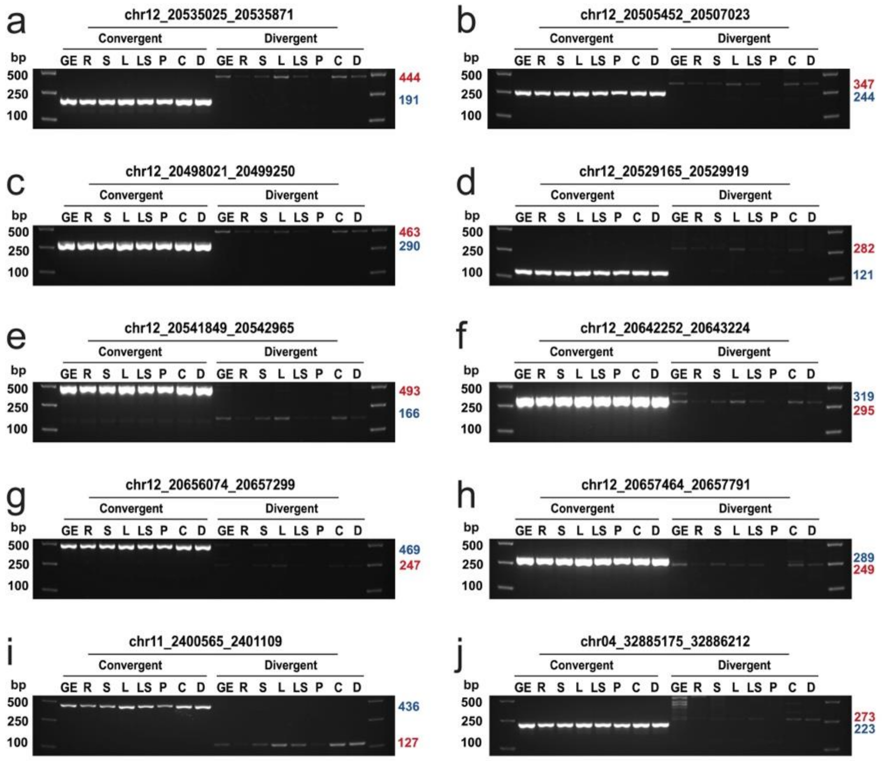

Supplementary Fig. 3. PCR validation of ten eccDNAs with direct repeats.

Validated eccDNAs with direct repeats are indicated for: (a) chr12_20535025_20535871, (b) chr12_20505452_20507023, (c) chr12_20498021_20499250, (d) chr12_20529165_20529919, (e) chr12_20541849_20542965, (f) chr12_20642252_20643224, (g) chr12_20656074_20657299, (h) chr12_20657464_20657791, (i) chr11_2400565_2401109, (j) chr04_32885175_32886212. Source data are provided as a Source Data file.

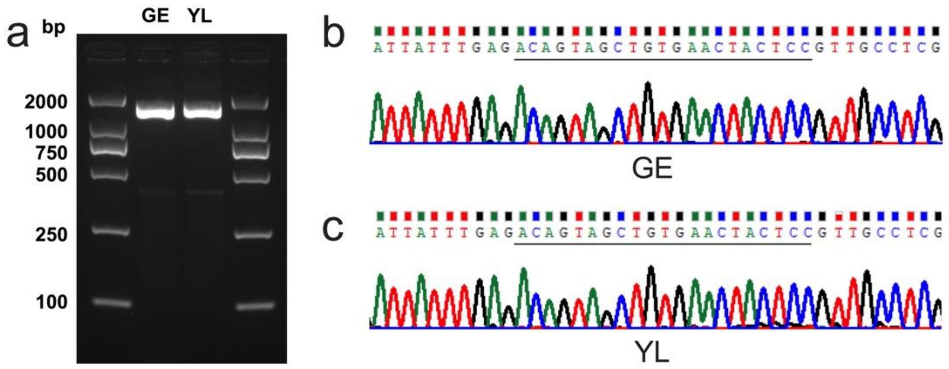

Supplementary Fig. 4. Pre-optimization detection and sequencing of chromosomal microdeletion. (a) This agarose gel image displays PCR products before optimization, targeting a chromosomal microdeletion within the range of chr12:20609533-20610693. The lanes are marked with the expected sizes for both deleted and non-deleted chromosomal forms. DNA was extracted from two types of tissues, germinated embryo (GE) and young leaf (YL), which are indicated above the respective lanes. (b) Sequencing chromatogram of the PCR product from the GE tissue, showing the sequence corresponding to the deleted chromosome with a direct repeat underlined, illustrating the genomic alteration. (c) Similar to (b), this chromatogram represents the PCR product from YL tissue, also indicating the deletion with the remaining direct repeat sequence underlined, confirming the consistency of the microdeletion across different tissues. Source data are provided as a Source Data file.

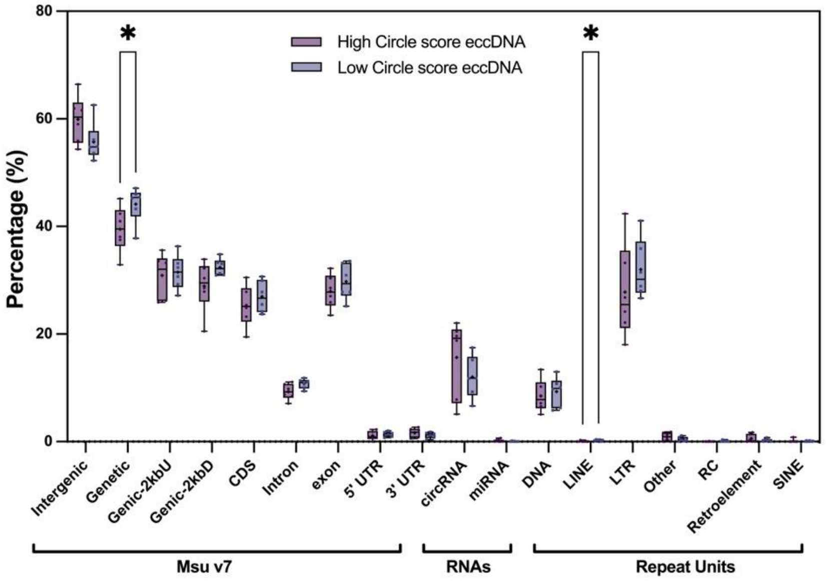

Supplementary Fig. 5. Distribution of extrachromosomal circular DNA (eccDNA) by circle score categories in different genomic regions. This box plot represents the distribution of eccDNAs categorized by their Circle Scores, with two distinct groups: 'High Circle Score eccDNAs' (score > 100,000) indicated in dark grey, and 'Low Circle Score eccDNAs' in light grey. The plot quantitatively compares the percentage of eccDNAs originating from various genomic regions such as intergenic spaces, coding sequences (CDS), introns, untranslated regions (UTRs), and different types of RNA and repeat units. The data are presented as individual points. Notably, 'Genetic' and 'LINE' categories show significance with *p*-values of 0.041126. The two-tailed Mann-Whitney test was used for significance testing, with results indicated by asterisks for levels where $p < 0.05$. Source data are provided as a Source Data file.

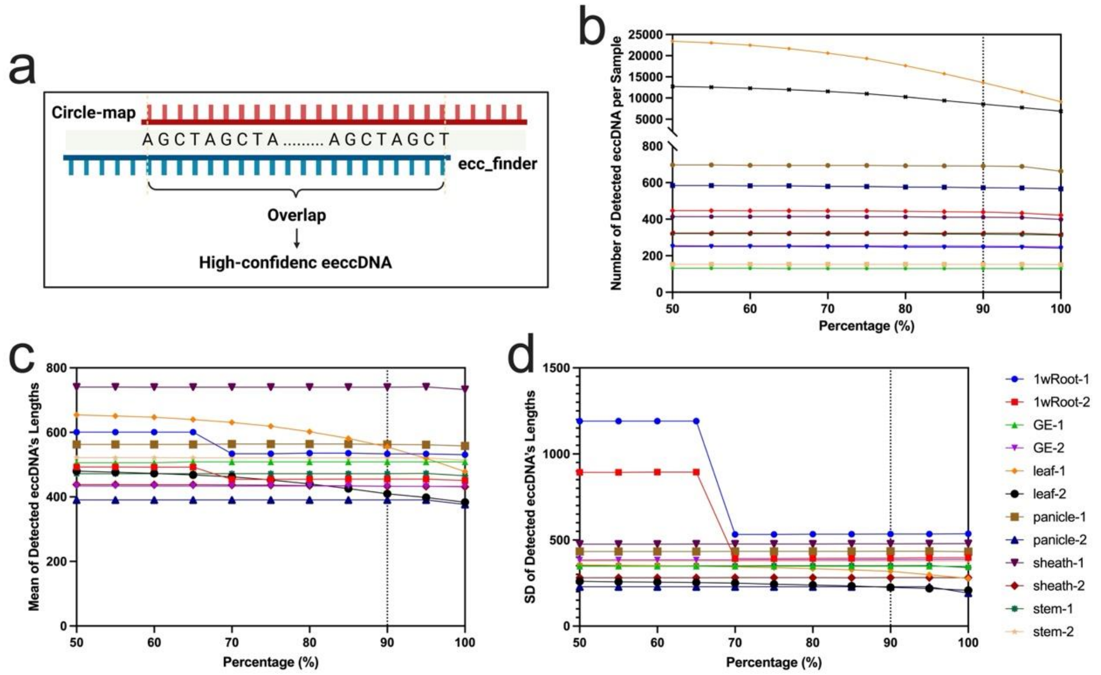

Supplementary Fig. 6. Multi-faceted eccDNA characterization and quantification. (a) Schematic representation of the eccDNA identification method combining two software tools, highlighting the overlap between Circle-map and ecc_finder to determine high-confidence eccDNA. (b) Depicts the number of detected eccDNAs per sample across various overlapping region thresholds from 50% to 100%. This graph shows a general decrease in detected eccDNA counts with increasing overlap percentage, identifying an optimal threshold for reliable eccDNA analysis. (c) Displays the mean lengths of detected eccDNAs, which remain relatively stable across the overlapping region thresholds, suggesting consistent size characterization of eccDNA through the range of overlap percentages. (d) Shows the standard deviation (SD) of detected eccDNA lengths, indicating the variability in eccDNA size decreases as the overlap threshold increases, further supporting the selection of a higher overlap percentage for accurate eccDNA characterization. Source data are provided as a Source Data file.

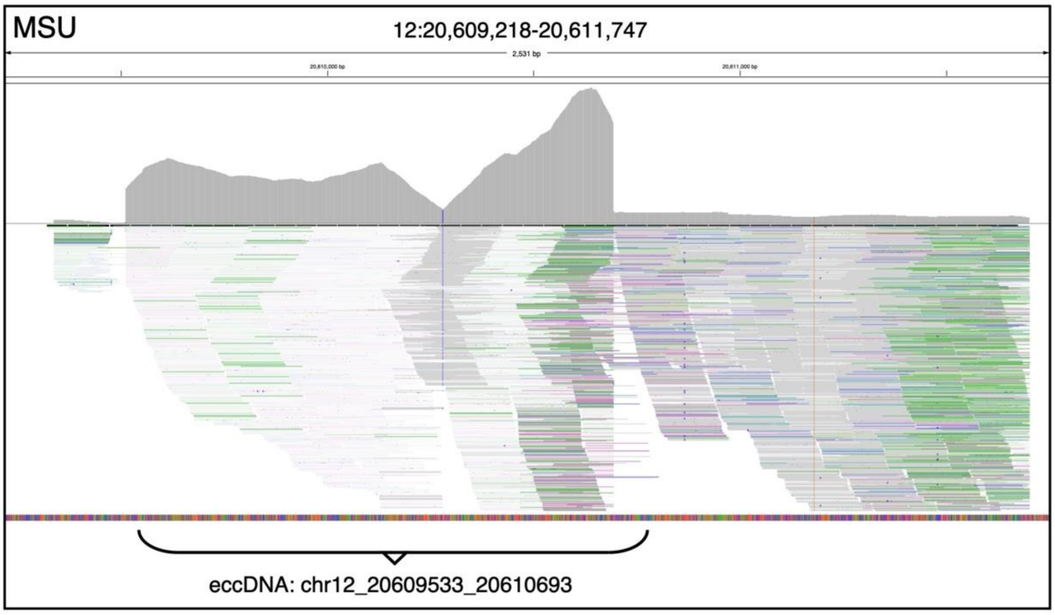

Supplementary Fig. 7. Visualization of read coverage at detected eccDNA loci using the Integrative Genomics Viewer (IGV).

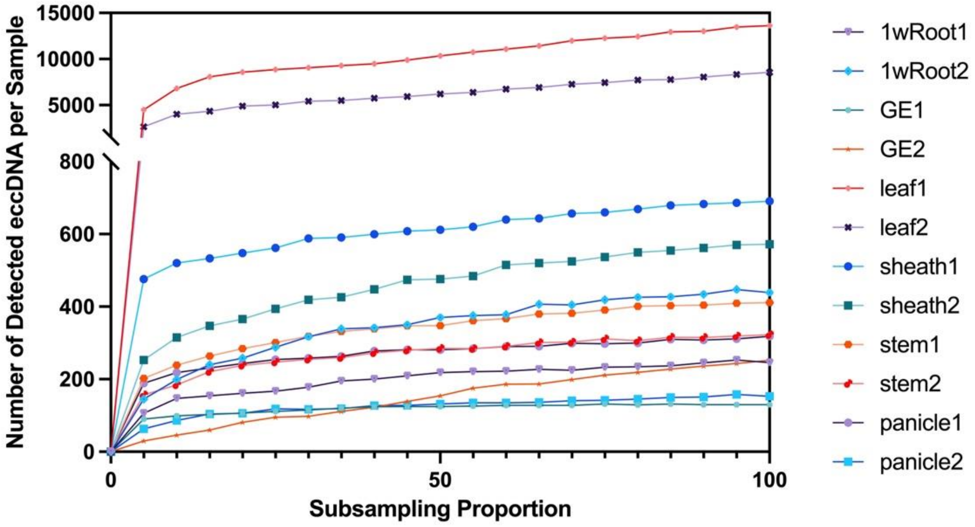

Supplementary Fig. 8. Saturation curve analysis of eccDNA detection. This figure illustrates a saturation curve analysis depicting the number of detected eccDNAs per sample at various subsampling proportions, from 0 to 100%. The curves plateau at different levels for each sample type, indicating the point at which further subsampling does not significantly increase the number of detected eccDNAs. This suggests a stable representation of eccDNAs in the subsamples and supports the efficacy of the extraction method for both high and low abundant eccDNA forms. Source data are provided as a Source Data file.

Supplementary Note 1. Comprehensive identification of extrachromosomal circular DNA in rice tissues

After implementing the circle-seq protocol outlined in Fig.1a on six distinct rice tissue samples, we generated approximately 28 billion high-quality, paired-end reads.

In analyzing the sequencing data, we applied two methodologies (Supplementary Fig. 6a). Initially, utilizing the circle-seq software[1], we conducted a preliminary identification and analysis of eccDNAs within the dataset. The subsequent filtering applied criteria including: (1) presence of at least one discordant read pair and a minimum of two independent split reads verifying breakpoint coordinates; (2) a circle score above 50, reflecting alignment quality and split read support for the circular DNA; (3) a coverage ratio between 0.33 and 1 at breakpoints, indicating high-quality circles; and (4) zero coverage discontinuity, ensuring complete read coverage.

For each sample, we formulated a putative list of eccDNAs and subsequently amalgamated these lists for advanced analysis. Utilizing ecc_finder software (version 1.0.0)[2], we identified potential eccDNAs across samples. Verification involved intersecting these eccDNA lists using bedtools (version 2.29.2)[3], creating a unified eccDNA coordinate list. To affirm our findings, we established an overlap region with gradations from 50% to 100%, calculating the average quantity and length of eccDNAs within each, alongside their standard deviations (SD).

Supplementary Fig.6b-d illustrate that the average eccDNA length stabilizes above the 90% overlap threshold, suggesting size consistency within this range. However, the eccDNA count declines significantly at higher thresholds, particularly beyond 90%, indicating that increasing specificity may exclude many shorter eccDNAs. Moreover, the standard deviation in eccDNA length decreases markedly at thresholds over 90%, reflecting greater length uniformity at these specificities. Considering both eccDNA

length and quantity, we selected an overlap threshold above 90%.

In this strategy, we identified a total of 25,598 eccDNAs across six tissues (Fig. 2a). For validation, we randomly chose an eccDNA (chr12_20609533_20610693), extracting its region's reads from the dataset (Supplementary Fig.7). This confirmed the identified eccDNA's reliability through concordant read mappings across the region, indicative of a circular DNA structure. Specifically, alignments showed dense reads with split alignments and discordant pairs matching the anticipated breakpoints, verifying the DNA's circular nature. Further PCR validation on 10 randomly chosen eccDNAs corroborated our method's reliability (Supplementary Fig. 3).

And then, in our efforts to analyze eccDNA across various rice tissues, we conducted a thorough saturation analysis for each sample. Guided by techniques described in the literature[4], we adopted a strategy of 5% incremental subsampling, a process detailed in Supplementary Fig.8. The resulting saturation curves for each sample and replicate, as depicted in the accompanying figure, exhibit a definitive trend towards saturation. This indicates that our methodology is effective in capturing a comprehensive profile of eccDNA circles present in the samples.

We observed variations in eccDNA circle scores across rice tissues, with leaves displaying many low score eccDNAs (Figure 2d). This may suggest either a technical issue with the software or an experimental procedure aspect needing further investigation. Currently, we hypothesize this phenomenon to be unique to rice leaves, possibly due to their high eccDNA quantity. The specific reasons behind this trend require additional analysis, but we believe it does not detract from our method's effectiveness in identifying eccDNA.

Supplementary references

1 Prada-Luengo, I., Krogh, A., Maretty, L. & Regenberg, B. Sensitive detection of circular DNAs at single-nucleotide resolution using guided realignment of partially aligned reads. *BMC Bioinformatics* **20**, 663 (2019).

2 Zhang, P., Peng, H., Llauro, C., Bucher, E. & Mirouze, M. ecc_finder: A robust and accurate tool for detecting extrachromosomal circular DNA from sequencing data. *Frontiers in Plant Science* **12** (2021).

3 Quinlan, A. R. & Hall, I. M. BEDTools: A flexible suite of utilities for comparing genomic features. *Bioinformatics* **26**, 841-842 (2010).

4 Prada-Luengo, I. *et al.* Replicative aging is associated with loss of genetic heterogeneity from extrachromosomal circular DNA in *Saccharomyces cerevisiae*. *Nucleic Acids Research* **48**, 7883-7898 (2020).
