# Supplementary material for: Dynamics of extrachromosomal circular DNA in rice
Source: Nat Commun. 2024 Mar 18;15:2413. doi: 10.1038/s41467-024-46691-0 (PMC10948907; doi:10.1038/s41467-024-46691-0)
Supplement: Supplementary file 3 — Description of Additional Supplementary Files [file 41467_2024_46691_MOESM3_ESM.pdf]

Description of Additional Supplementary Files

Supplementary Data 1. Summary of eccDNA sequencing in rice tissues.

Supplementary Data 2. Comprehensive list of eccDNAs in various rice tissues.

Supplementary Data 3. Unique eccDNAs across rice tissue types.

Supplementary Data 4. Ubiquitous eccDNAs identified in rice tissues.

Supplementary Data 5. Distribution of ubiquitous and unique eccDNA in rice tissues.

Supplementary Data 6. eccDNAs identified in five additional sample groups.

Supplementary Data 7. Organelle-specific eccDNAs in rice tissues.

Supplementary Data 8. Primer sequences for PCR confirmation of eccDNA presence.

Supplementary Data 9. Comprehensive list of eccDNA-associated genes across rice tissue samples.

Supplementary Data 10. Gene expression levels (TPM) annotated by MSU v7 for all genes.
