# Supplementary material for: Dynamics of extrachromosomal circular DNA in rice
Source: Nat Commun. 2024 Mar 18;15:2413. doi: 10.1038/s41467-024-46691-0 (PMC10948907; doi:10.1038/s41467-024-46691-0)
Supplement: Supplementary file 14 — Reporting Summary [file 41467_2024_46691_MOESM14_ESM.pdf]

nature portfolio

Corresponding author(s): Bin Han & Tingting Lu

Last updated by author(s): Mar 2, 2024

Reporting Summary

Nature Portfolio wishes to improve the reproducibility of the work that we publish. This form provides structure for consistency and transparency in reporting. For further information on Nature Portfolio policies, see our Editorial Policies and the Editorial Policy Checklist.

Statistics

For all statistical analyses, confirm that the following items are present in the figure legend, table legend, main text, or Methods section.

n/a	Confirmed	
☐	☒	The exact sample size (*n*) for each experimental group/condition, given as a discrete number and unit of measurement
☐	☒	A statement on whether measurements were taken from distinct samples or whether the same sample was measured repeatedly
☐	☒	The statistical test(s) used AND whether they are one- or two-sided<br>*Only common tests should be described solely by name; describe more complex techniques in the Methods section.*
☐	☒	A description of all covariates tested
☐	☒	A description of any assumptions or corrections, such as tests of normality and adjustment for multiple comparisons
☐	☒	A full description of the statistical parameters including central tendency (e.g. means) or other basic estimates (e.g. regression coefficient) AND variation (e.g. standard deviation) or associated estimates of uncertainty (e.g. confidence intervals)
☐	☒	For null hypothesis testing, the test statistic (e.g. *F*, *t*, *r*) with confidence intervals, effect sizes, degrees of freedom and *P* value noted<br>*Give P values as exact values whenever suitable.*
☐	☒	For Bayesian analysis, information on the choice of priors and Markov chain Monte Carlo settings
☒	☐	For hierarchical and complex designs, identification of the appropriate level for tests and full reporting of outcomes
☐	☒	Estimates of effect sizes (e.g. Cohen's *d*, Pearson's *r*), indicating how they were calculated

Our web collection on statistics for biologists contains articles on many of the points above.

Software and code

Policy information about availability of computer code

Data collection	No software was used.
Data analysis	In this study, the principal tools utilized for the analysis and identification of extrachromosomal circular DNA (ecDNA) were Circle-Map (version 1.1.4), ecc_finder (version 1.0.0), and bedtools (version 2.29.2). Additional software applications and their corresponding versions are detailed in the Methods section and are available in our dedicated GitHub repository: [https://github.com/YxZhang-XHCY/eccToolkit].

For manuscripts utilizing custom algorithms or software that are central to the research but not yet described in published literature, software must be made available to editors and reviewers. We strongly encourage code deposition in a community repository (e.g. GitHub). See the Nature Portfolio guidelines for submitting code & software for further information.

Data

Policy information about availability of data

All manuscripts must include a data availability statement. This statement should provide the following information, where applicable:

- Accession codes, unique identifiers, or web links for publicly available datasets
- A description of any restrictions on data availability
- For clinical datasets or third party data, please ensure that the statement adheres to our policy

We have ensured that the manuscript clearly addresses this requirement in the designated sections for Data Availability and Code Availability.

Research involving human participants, their data, or biological material

Policy information about studies with human participants or human data. See also policy information about sex, gender (identity/presentation), and sexual orientation and race, ethnicity and racism.

Reporting on sex and gender	n/a
Reporting on race, ethnicity, or other socially relevant groupings	n/a
Population characteristics	n/a
Recruitment	n/a
Ethics oversight	n/a

Note that full information on the approval of the study protocol must also be provided in the manuscript.

Field-specific reporting

Please select the one below that is the best fit for your research. If you are not sure, read the appropriate sections before making your selection.

☒ Life sciences ☐ Behavioural & social sciences ☐ Ecological, evolutionary & environmental sciences

For a reference copy of the document with all sections, see nature.com/documents/nr-reporting-summary-flat.pdf

Life sciences study design

All studies must disclose on these points even when the disclosure is negative.

Sample size	In determining the sample size for our study, we referenced prior research within our field that utilized similar experimental setups, aiming to balance scientific rigor with practical feasibility. Considering the variability observed in preliminary experiments and aiming for sufficient statistical power to detect meaningful differences across conditions, we chose to use two replicates for each of the eight distinct sample categories, resulting in a total sample size of sixteen. For the supplementary study on rice leaf tissues under varying conditions, three replicates were used to enhance the reliability of our findings in these comparisons. This approach aligns with common practices in eccDNA research, ensuring that our study's scale is adequate to capture significant biological effects while acknowledging the constraints of our experimental resources.
Data exclusions	All data collected were incorporated into our analyses, ensuring comprehensive representation and rigorous evaluation.
Replication	Due to the inherent randomness in the formation of eccDNA, only a fraction of our attempts were successful. However, each sample type was repeated twice to increase the chances of capturing the phenomenon.
Randomization	The samples were not allocated to experimental groups randomly. Instead, samples were categorized based on their biological tissue type or treatment condition. Thus, the same type of tissue or treated sample was grouped together.
Blinding	Throughout the experimental and analysis stages, researchers were able to identify the different sample groups due to their origins from distinct biological tissues or different treatment conditions. In this context, implementing blinding in the traditional sense was not feasible. However, to minimize bias and subjective judgment's impact on the study's outcome as much as possible, we adopted alternative measures to ensure the objectivity and reliability of the results. These measures included using standardized protocols for data collection and analysis, and where possible, having the data reviewed by independent researchers. We believe that, although blinding was not implemented, these steps are sufficient to ensure the fairness of the study and the accuracy of its findings.

Reporting for specific materials, systems and methods

We require information from authors about some types of materials, experimental systems and methods used in many studies. Here, indicate whether each material, system or method listed is relevant to your study. If you are not sure if a list item applies to your research, read the appropriate section before selecting a response.

Materials & experimental systems

n/a	Involved in the study
x	Antibodies
x	Eukaryotic cell lines
x	Palaeontology and archaeology
x	Animals and other organisms
x	Clinical data
x	Dual use research of concern
	x Plants

Methods

n/a	Involved in the study
x	ChIP-seq
x	Flow cytometry
x	MRI-based neuroimaging

Dual use research of concern

Policy information about dual use research of concern

Hazards

Could the accidental, deliberate or reckless misuse of agents or technologies generated in the work, or the application of information presented in the manuscript, pose a threat to:

No	Yes
x	Public health
x	National security
x	Crops and/or livestock
x	Ecosystems
x	Any other significant area

Experiments of concern

Does the work involve any of these experiments of concern:

No	Yes
x	Demonstrate how to render a vaccine ineffective
x	Confer resistance to therapeutically useful antibiotics or antiviral agents
x	Enhance the virulence of a pathogen or render a nonpathogen virulent
x	Increase transmissibility of a pathogen
x	Alter the host range of a pathogen
x	Enable evasion of diagnostic/detection modalities
x	Enable the weaponization of a biological agent or toxin
x	Any other potentially harmful combination of experiments and agents

Plants

Seed stocks	The rice seeds we used are the standard 'Nipponbare' variety, stored in the seed bank of the NCGR.
Novel plant genotypes	n/a
Authentication	n/a
